# Impaired Dynamics of Positional and Contextual Neural Coding in an Alzheimer’s Disease Rat Model

**DOI:** 10.3233/JAD-231386

**Published:** 2024-08-27

**Authors:** Athira Nataraj, Annu Kala, Stephanie Lissette Proskauer Pena, Karel Jezek, Karel Blahna

**Affiliations:** Biomedical Center, Faculty of Medicine in Pilsen, Charles University, , Prague, Czech Republic

**Keywords:** Alzheimer’s disease, place cell directionality, place field size, spatial memory, TgF344-AD rats

## Abstract

**Background:**

The hippocampal representation of space, formed by the collective activity of populations of place cells, is considered as a substrate of spatial memory. Alzheimer’s disease (AD), a widespread severe neurodegenerative condition of multifactorial origin, typically exhibits spatial memory deficits among its early clinical signs before more severe cognitive impacts develop.

**Objective:**

To investigate mechanisms of spatial memory impairment in a double transgenic rat model of AD.

**Methods:**

In this study, we utilized 9–12-month-old double-transgenic TgF344-AD rats and age-matched controls to analyze the spatial coding properties of CA1 place cells. We characterized the spatial memory representation, assessed cells’ spatial information content and direction-specific activity, and compared their population coding in familiar and novel conditions.

**Results:**

Our findings revealed that TgF344-AD animals exhibited lower precision in coding, as evidenced by reduced spatial information and larger receptive zones. This impairment was evident in maps representing novel environments. While controls instantly encoded directional context during their initial exposure to a novel environment, transgenics struggled to incorporate this information into the newly developed hippocampal spatial representation. This resulted in impairment in orthogonalization of stored activity patterns, an important feature directly related to episodic memory encoding capacity.

**Conclusions:**

Overall, the results shed light on the nature of impairment at both the single-cell and population levels in the transgenic AD model. In addition to the observed spatial coding inaccuracy, the findings reveal a significantly impaired ability to adaptively modify and refine newly stored hippocampal memory patterns.

## INTRODUCTION

Alzheimer’s disease (AD) is a progressive neurodegenerative disorder and is currently the leading cause of dementia worldwide.^1–3^ Pathological characteristics of AD include the formation of amyloid-β plaques, intracellular formation of hyperphosphorylated tau accompanied by neuroinflammation, and neuronal loss.^2,4–7^ Clinically, the prominent early sign of AD is a major learning and memory deficit that progresses into an overall deterioration of cognitive and other brain functions. There are two basic etiological forms of AD: familial, which is rather rare (6–10%) and marked by inherited gene mutation with an early onset, and sporadic, which develops in older age with multifactorial etiology.^8,9^ Various transgenic rodent models (such as TgAPPswe, Tg6590, APP21 and APP31, TgF344-AD) were developed to better understand the pathophysiology of AD and to design and test potential therapeutic strategies.^10–13^

In this study, we employed the TgF344-AD rat model, recognized as one of the prominent models for AD, to explore the mechanisms behind spatial learning and memory deficits associated with AD. The model carries two human gene mutations implicated in AD: human amyloid-β precursor protein (AβPP) with the Swedish mutation and human presenilin (PSEN) 1 with Δ exon 9 mutations. Despite the minimalistic genetic modification, TgF344-AD rats exhibit a complete repertoire of AD pathological features in an age-dependent manner, with a combination of amyloid plaques, the formation of hyperphosphorylated tau tangles, and memory impairment. The model was developed on a Fisher344 background, a rat strain with accelerated aging properties widely used in aging research. This leads to an early onset of memory deficits in TgF344-AD rats.^14–16^

The impact of transgenes carried by TgF344-AD on memory has been characterized by numerous studies since the introduction of the model. Cohen et al. (2013), in their seminal study, showed impaired spatial memory in the Barnes maze task at 15 and 25 months of age.^16^ A later study reported age-dependent impairments in spatial navigation tested by the Morris water task.^17^ More recent studies identified the onset of spatial memory impairment around the age of 4–5 months.^14,15^ Besides spatial memory and navigation, TgF344-AD rats exhibit age-dependent changes in anxiety level estimates, motor activity, and alterations in olfactory and visual functions.^18^ Apart from cognitive impairments, various neural network disturbances were reported in AD transgenic models. Earlier studies showed disrupted hippocampal theta oscillation and decreased theta-phase gamma-amplitude coupling in aged TgF344-AD.^19^

The activity of populations of hippocampal place cells is considered to constitute a neural substrate for spatial memory. Place cells, found in hippocampal subfields CA1–3, are pyramidal cells whose activity is strongly influenced by an animal’s position and the surrounding context, resulting in distinct cell-specific zones called firing fields. Consequently, populations of place cells form context-specific neural representations of familiar environments. Studies have indicated an impact on the hippocampal neural code for space in TgF344-AD rats. For instance, Galloway et al. documented a reduction in spatial coding accuracy in CA3 and CA2 regions among 12–20 months old TgF344-AD rats.^20^ Another study focusing on 18–20 months old TgF344-AD rats reported dysfunctional spatial tuning concerning spatial information content, stability, and in-field firing rates.^21^ However, these findings primarily focus on age levels beyond one year, which represent a rather highly advanced stage in the progression of AD-related pathology. Consequently, data describing the development of spatial coding deficits at stages prior to 12 months are lacking.

In the present study, our goal was to thoroughly characterize changes in neural coding of space within the hippocampal CA1 region in the TgF344-AD model at the age of 9–12 months. We focused on the hippocampal code alterations under both familiar, well-known conditions and during the formation of a novel hippocampal map. We utilized a one-dimensional linear maze because it induces stereotypical behavior, making it well-suited for sensitively measuring changes in the precision of spatial coding, the development of spatial representation in a novel environment, and its directional characteristics. The latter feature is considered to transcend spatial coding in a Cartesian coordinate system and provides an additional dimension of expectation into the hippocampal positional activity. Hippocampal place cells exhibit place-specific firing either only in one direction (unidirectional cells) or in both forward and reverse directions (bidirectional cells).^22^ Across the place cell population, the directionality of place fields is a subject of development—initially less expressed, with experience, the activity gradually becomes more direction-specific.^23^ These findings suggest elements of contextual coding in hippocampal spatial information processing that gradually develop with experience. We understand the development of place cell directionality as a unique kind of memory maturation, a consolidation in a broader sense of meaning. Such experience-dependent evolution of information content has not been studied in relation to AD models.

Among other changes, we observed a decreased proportion of unidirectional firing in both familiar and novel environment conditions in TgF344-AD rats compared to controls. The rate of directionality development was also found to be significantly slower in transgenics than in controls. Overall, our study indicates an impairment in the amount of spatial information carried by the hippocampal cell population and a deteriorated capacity for encoding novel information in the AD model. Moreover, it suggests a slowed and impaired ability to incorporate aspects of contextual information within the CA1 hippocampal memory code.

## METHODS

### Subjects

Experiments were performed using a total of 10 male rat subjects aged 9–12 months, divided into two groups. One group consisted of TgF344-AD (Tg) rats and their age-matched wild-type littermates served as control (Ctrl) counterparts. TgF344-AD subjects expressed two mutations associated with the familiar form of AD, human amyloid-β precursor protein (AβPP) with Swedish mutation and human presenilin (PSEN) 1 with Δ exon 9 mutations.^16^ Genotyping was performed by qualitative PCR followed by gel electrophoresis. The primers used were APP (Forward: 5^′^- CCG AGA TCT CTG AAG TGA AGA TGG ATG- 3^′^) and PS1 (Forward: 5^′^-CAG GTG GTG GAG CAA GAT G- 3^′^). All protocols followed in this study were approved by the Ethical Committee of the Ministry of Education, Youth and Sports of the Czech Republic (approval no. MSMT-12048/2019-14) according to the Guide for the Care and Use of Laboratory Animals (Protection of Animals from Cruelty Law Act No. 246/92, Czech Republic). The animals were housed and maintained on a 12-h light/12-h dark cycle.

### Vision test

The vision of experimental subjects was assessed using the Morris Water Maze (MWM) protocol. The MWM consisted of a white plastic circular tank that measured 180 cm in diameter and 60 cm in height, filled with water at a temperature of 20°C and a depth of 40 cm. Inside the tank, a metallic visible platform with a diameter of 15 cm was placed 0.5 cm above the water’s surface in the southwestern quadrant, secured by a 25 cm-high green bar. Rats were released from four different starting points (N, S, E, W) facing the wall, and the time it took for them to reach the platform was measured. Typically, the rats found the goal within 60 s. If a rat failed to reach the platform within this time frame, the experimenter guided the animal to the platform. In either case, once the rat reached the platform, it was given a 30-s pause before being transferred to a waiting cage. This procedure was repeated by releasing the animals from three other different sides into the maze, and their vision was recorded on two days of testing. Animals that failed to locate the platform within 60 s in more than two trials on the second day were excluded from the study.

### Experimental procedure

All subjects underwent a pre-training phase on a 1D linear track for 14 days before microdrive implantation. The linear track, which measured 170 cm in length, was enclosed by high walls on both sides and featured several proximal and distal cues such as LED lights, and a differently textured track floor. During the training sessions, rats shuttled back and forth along the track to collect rewards placed at both ends for approximately 20–30 min per day. After the pre-training phase, electrode implantation surgery was performed (detailed below), and the animals were given a week to recover. Training sessions continued for an additional 10–14 days, during which the tetrodes were slowly positioned into the hippocampal CA1 pyramidal layer. On the day of the experiment, the animals were allowed to rest in the recording chamber for 1 h. Then, the session of approximately 30 min of back-and-forth runs in a familiar (fam) environment was recorded. After another 1-h break, a novel (nov) environment with different cues was introduced, and another 30 min of hippocampal activity was recorded. Our experimental design, aimed at acquiring runs within a 30-min timeframe, results in a variable number of runs for each group. Information on the number of runs for all animals in both groups is provided in the Supplementary Table 1. The position of the LEDs on the head preamplifier was used to track the animal’s position.

### Microdrive implantation

The animals were anesthetized with isoflurane (4% for induction and 1–1.5% for maintenance) while receiving a 1.5% oxygen flow. Additionally, dexmedetomidine and nalbufin (0.5 ml/kg and 1 ml/kg, respectively) were administered intraperitoneally. The rats were then fixed in a stereotaxic frame. A hyperdrive, which held 16 individually movable tetrodes, was implanted above the hippocampal region at coordinates (AP-3.8 mm, ML-3.2 mm). Each individual tetrode was composed of four twisted 17*μ*m polyimide-coated platinum-iridium wires, and the impedance was reduced to approximately 210 k*Ω* by electroplating with platinum. Once the drive was securely fixed in the skull using 6–8 stainless steel bone screws and dental acrylic, the animals were gradually brought out of isoflurane anesthesia and received post-surgery care medications, including carprofen (Rimadyl, 1 ml/kg) and marbofloxacin (Marbocyl, 0.5 ml/kg).

### Spike sorting and position monitoring

Electrophysiological data were acquired and recorded using a computer-based data acquisition system, Axona (DacqUSB). Neuronal activity was amplified and band-pass filtered. Raw signals were recorded at 24 KHz and later subsampled to 20 KHz. Action potentials were extracted by first calculating power in the 800–9000 Hz range within a sliding window (12.8 ms), and further action potentials with power higher than 5 standard deviations from the baseline mean were selected. The process of spike detection from the local field potential and sorting was carried out in a manner similar to a previous study.^24^ Spike features were extracted using principal component analysis, and action potentials were separated into multiple single units using semi-automatic clustering software (http://klustakwik.sourceforge.net/).^25^ Clusters were then manually reviewed and corrected using a graphical cluster-cutting program, Sgclust (©Jozsef Csicsvari). Units were classified based on well-defined cluster boundaries and clear refractory periods in their autocorrelations. Pyramidal cells and interneurons in the CA1 region were distinguished based on firing rate, autocorrelations, and waveforms.

### Histology

**Fig. 1:**
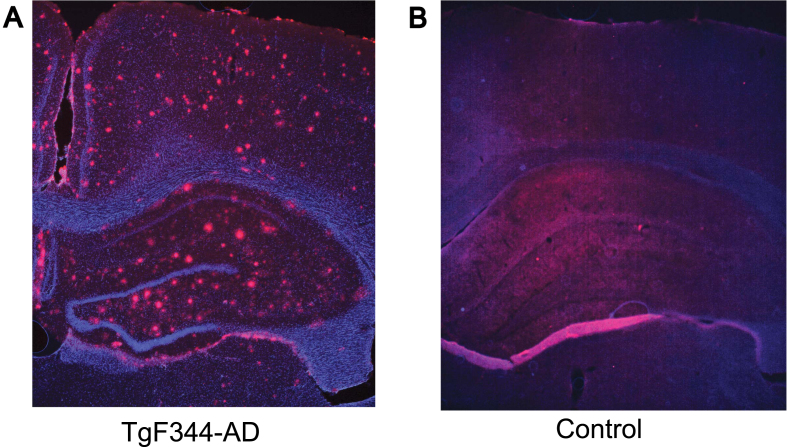
Hippocampal pathology in TgF344-AD rats (9–12 months). Representative images of amyloid-β (red) and DAP1 (blue) staining in 9–12 months old TgF344-AD (A) and control (B) rat. The coronal section from a transgenic rat (A) shows the accumulation of amyloid-β plaques throughout the hippocampus and cortex, while the control animal (B) exhibits no apparent pathology.

After acquiring electrophysiological data, the rats were sacrificed, and intracardial perfusion of the brain was performed using 4% paraformaldehyde and Ringer’s Solution. Brain tissue was then immersed in 30% sucrose, and 50*μ*m thick sections were obtained using a microtome. These sections were taken at the level of the hippocampus. After blocking the sections with TBS block solution and washing with 1XTBS, the sections were probed with an anti-beta amyloid primary antibody (Abcam beta amyloid-ab2539) at a 1:200 dilution overnight at 4°C. The sections were subsequently washed, and they were then incubated with a goat anti-rabbit secondary antibody (Invitrogen Alexa FluorTM Plus 647-A32733) at a dilution of 1:1000 for 2–3 h at room temperature in the dark. Further examination using a fluorescence microscope confirmed the presence of amyloid beta plaques in TgF344-AD rats but not in their control counterparts (Fig. 1). To confirm the tetrode positioning in the hippocampal CA1 layer, a Nissl staining protocol was performed, and the sections were examined under a bright field microscope.

### Data analysis

#### Position data

One-dimensional position data were extracted from two-dimensional spatial coordinates on the linear track. The animal’s instantaneous speed was calculated from 2D data and smoothed with a Hamming window with a length of 0.3 s. Data in which animals moved at speeds greater than 3 cm/s were only included in the analysis, while trajectories with lower speeds and reward consumption area, 9 cm from both ends of the linear track, were excluded. This exclusion was aimed to effectively eliminate potential artifacts (such as sharp-wave ripple activity with higher firing patterns) during periods of reduced animal activity. For further analysis, the position on the linear track was subdivided into 3 cm bins (a total of 57 bins on the 170 cm linear track).

### Place field analysis

**Fig. 2:**
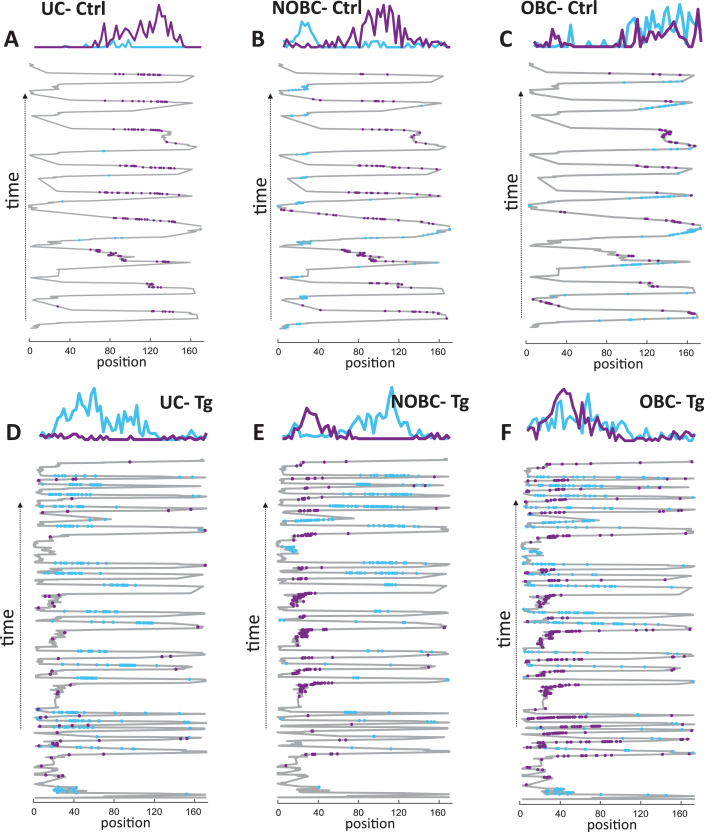
Place cell firing rate profile in control and TgF344-AD rats. Examples of a unidirectional cell (UC), a non-overlapping bidirectional cell (NOBC), and an overlapping bidirectional cell (OBC) from a single animal are illustrated from both the control group (A–C) and from the transgenic group (D–F) across the running trials. The criteria of UC, NOBC, and OBC are described in the methods section. In each plot, the upper panel displays firing rate profiles, while the lower panel shows the spike distribution of place cells projected on the x coordinate of the trajectory across time on a 1-D linear track. Place cell’s firing in the forward direction is represented in blue, and in the reverse direction in violet.

For the comparison of single-cell activity between control and TgF344-AD animals, the entire set of pyramidal cells with an average firing rate across both sessions in the range of 0.1–6 Hz, autocorrelations with a refractory period of 1–2 ms and stable spike projection principal components across the entire recording time were selected. Firing rate profiles were calculated for each 3 cm bin during active periods. The firing rate profiles of three different types of place cells (UC, NOBC, OBC) from both a control and a transgenic animal were illustrated in Fig. 2. In the upper panel, three figures depict, each representing one distinct type of place cell from a control animal, while the lower panel shows three figures, each representing a type of place cell from a Tg animal. Despite the dissimilar number of runs between the groups, the normalization of average values of individual spikes over time ensures that the varying number of runs between the groups does not affect the comparisons of place cells.

Firing rates were assessed by dividing the total number of spikes by time. To evaluate the overall firing response from the familiar to the novel environment, we further analyzed a normalized firing rate for each cell throughout all running laps across both sessions. This involved normalizing firing rates with respect to the maximum value observed for eachcell.

### Spatial information

Spatial information (SI) was computed between spike trains within each bin and the animal’s current position, corrected for a finite sampling size.^26,27^

$$\mathrm{SI}(\mathrm{bit}/\mathrm{spike})=\sum_{i=1}^{N}\mathrm{Pi}(\mathrm{Ri}/R)\log2(\mathrm{Ri}/R)$$

Where i was the bin ID number, Pi was the probability for occupancy of bin I, Ri was the mean firing rate for bin i and R was the overall firing mean rate.

### Place field size

The area under the upper 80% of the firing rate profile was included for metric analysis. The length between the border points projected under this area was defined as the place field size.

### Directional activity of place cells

Calculations for the directional characteristics of place cells were performed using the modified algorithm from a previous study to capture the exact overlap of firing rate profiles across both directions.^22^ Firstly, place field profiles were estimated as a result of the average firing rate for each spatial N_bins_ and consequently for cells with peak of minimum 1.2 Hz for both directions. The overlap index r value between place field profiles P (*φ*) from both directions was calculated with the formula:

$$r=\frac{2\sum_{1}^{\varphi }\min(P\;\mathrm{right}\;(\varphi ),\;P\;\mathrm{left}\;(\varphi )\min\;(P\;\mathrm{right}\;(\varphi ),\;P\;\mathrm{left}\;(\varphi ))}{\sum \left(P\;\mathrm{right}(\varphi ),\;P\;\mathrm{left}(\varphi )\right)}$$

$$\mathrm{Pright},\mathrm{left}(\varphi )=\frac{N\;\mathrm{bind}\;P\;\mathrm{right},\;\mathrm{left}(\varphi )}{\sum_{1}^{\varphi }\left(P\;\mathrm{right},\;\mathrm{left}(\varphi )\right)}$$

The r values range from 0 for non-overlapping place field profiles to 1 for profiles that were identical. Cells with an overlap index (OI) higher than 0.4 were classified as bidirectional overlapped cells, while cells with lower OI were classified as bidirectional non-overlapped cells. Cells exhibiting a single peak specific to a particular direction were classified as unidirectional cells. Other cells with peaks within 9 cm of rewarded areas or peaks smaller than 1.2 Hz were categorized among non-classified cells.

### Directionality index

To evaluate firing rate specificity for each direction, the directionality index (DI) was calculated in accordance with previous studies.^23,28^ The directionality index for each cell was determined by calculating the firing rate in each direction. The absolute value of the difference between firing rates for each direction was subsequently divided by the sum of the average firing rate. Since every alternate lap of the animal run represents one direction and obtained values of DI are calculated from their difference, data can be presented only as a number of trials. A DI = 0 indicated the identical firing pattern for both directions, while higher values of DI (up to 1) indicated cell directional specificity. DI values for each cell were calculated for every lap within both familiar and novel environments.

$$\mathrm{DI}(i)=\sum_{i=1}^{N}\left(\frac{\mathrm{abs}(\mathrm{DirA}(i)-\mathrm{DirB}(i))}{\mathrm{DirA}(i)+\mathrm{DirB}(i)}\right)$$

Where A and B indicated two different running directions.

### Population vector analysis

Population vector analysis described the average firing activity of the entire network for particular spatial bins.^23,29,30^ Firing rate profiles from each cell were first smoothed with a linear Gaussian filter with a standard deviation of 6 cm and normalized to a peak value of 1. The average firing rates from all cells were then stacked along the z-axis as vectors for each spatial bin. Each bin was Pearson correlated with other spatial bins for each direction. Hence, the population-level analysis compared the level of coding similarity between particular directions within familiar and novel mazes, as embodied in the correlation matrices. The maximal peaks from averaged off-diagonal areas were used for the final statistical comparison.

### Bootstrapping

The TgF344-AD and control groups each consisted of 5 animals, with a varying number of recorded neurons from each animal. Supplementary Table 2 shows the exact count of neurons for each individual. The natural variability in neurons occurs for each animal, and the neurons are subjected to multiple criteria, including stability, firing rate, and auto correlogram, to ensure the quality equivalence of cells. However, to eliminate the potential impact of sample size between the groups, a bootstrap analysis was performed. The transgenic cell population from both familiar and novel sessions was bootstrapped 10000 times, and the null hypothesis (H_0_) was estimated for spatial coding, place field size, and place cell proportion calculations. For spatial coding analysis, each bootstrapped sample (BS) mean value from the transgenic familiar/novel environment (*μ* BS Tg fam/nov) was subtracted from the corresponding mean value from the control familiar/novel environment (*μ* ctrl fam/nov). The proportion of transgenic BS values smaller than the control samples was used to estimate *p*-values. For place field proportion analysis, each *μ* BS Tg fam/nov was subtracted from the corresponding *μ* ctrl fam/nov, and the proportion of transgenic BS values greater than the control samples were used to estimate the *p*-values.

A similar procedure was employed for the comparison of firing rate and normalized firing rate within the transgenics, since the transgenics had a larger number of cells than controls. In this instance, we conducted 10000 bootstraps, sampling cells to the same number as in the control group. We then computed the difference between average values from the novel and familiar environments. The proportion of BS transgenic novel values greater than BS transgenic familiar values was used to estimate the *p*-values.

### Statistics

Comparisons were made using independent two-way ANOVA as well as regression analysis with the factors of the group (TgF344-AD×control) and session (familiar×novel), respectively. Where appropriate, a Chi-squared test was also performed. Values were reported as average±SEM unless stated otherwise.

## RESULTS

**Fig. 3:**
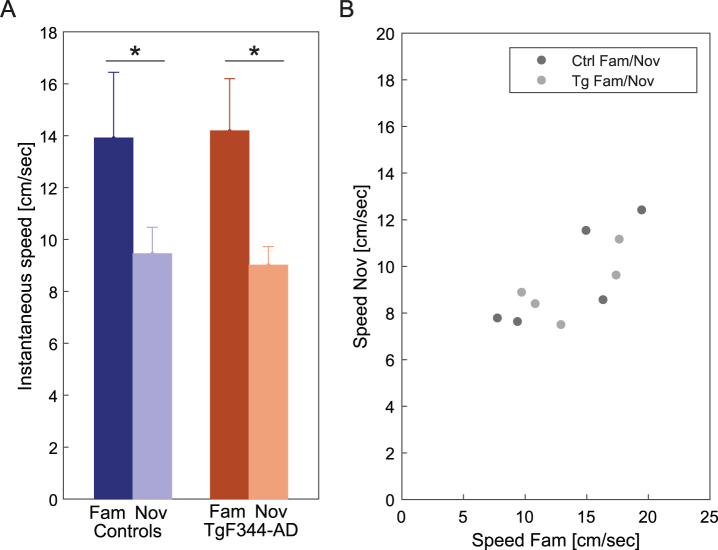
Instantaneous speed in familiar and novel environments. In panel A, a comparison of instantaneous speed (y-axis) between familiar and novel environments across groups (x-axis) reveals a significant decrease within both groups in the novel environment. In panel B, the plot illustrates the average speed of all animals across familiar and novel conditions. Each dot is the value of average speeds performed by one animal in a novel versus familiar environment (a total of 10 dots, 5 animals from each group). ^*^p < 0.05.

The instantaneous speed of both control and TgF344-AD rats during running trials on a linear track was assessed as a behavioral measure in response to familiar and novel environments. Both control and TgF344-AD groups exhibited significantly faster speed on the familiarized track compared to the novel track (Fig. 3A) [Ctrl: *n* = 5, fam: 13.9±5.0 cm/s; nov: 9.45±2.0 cm/sec; Tg: *n* = 5, fam: 14.2±4.0 cm/s; nov: 9.01±1.9 cm/sec]. An independent two-way ANOVA revealed a significant difference between the sessions [group effect: F (1, 16) = 0.003, *p* = 0.95; session effect: F (1, 16) = 9.667, *p* = 0.007; group*session effect: F (1, 16) = 0.053, *p* = 0.82]. The overall speed decline in a new environment reflects a reduced behavioral response to novelty. The average values from each animal in both groups were plotted against familiar and novel conditions. The average speed across the conditions followed a similar trend across both controls and transgenics (Fig. 3B). Each dot is the value of average speeds performed by one animal in a novel versus familiar environment (a total of 10 dots, 5 animals from each group).

To eliminate the impact of pauses between runs on the calculations of instantaneous speed and average firing rate between control and transgenic groups, we examined both the average number of stops during a single run and the average time spent on the track during the pause below the speed threshold for each group. No statistically significant difference was observed between the groups for both parameters. Detailed data is provided in Supplementary Figure 1.

### Increased average firing rate as a response to the novel condition

We analyzed the hippocampal network in both familiar and novel environments within both groups. Both controls and TgF344-AD rats exhibited a similar pattern of increased average firing rate in the novel condition [Ctrl cell no. = 174, fam: 1.18±0.09 Hz, nov: 1.5±0.12 Hz; Tg cell no. = 283, fam: 1.29±0.08 Hz; nov: 1.44±0.083 Hz]. A comparison of the average firing rate between the groups showed no significant difference by independent two-way ANOVA [group effect: F (1, 909) = 0.185, *p* = 0.66; session effect: F (1, 909) = 7.900, *p* = 0.005; group*session effect: F (1, 909) = 0.299, *p* = 0.585]. To address potential variations in sample sizes between the groups, bootstrap testing of average firing rates within transgenics has been included, given that transgenics had a larger number of cells. Average firing rate values from 10000 bootstrapped samples (BS) were calculated, with the majority (9,883 out of 10000) of values from the BS novel environments higher than values from the BS familiar environment (*μ* BS Tg nov > *μ* BS Tg fam = 0.16 Hz; *p* = 0.006) (Supplementary Figure 2A).

**Fig. 4:**
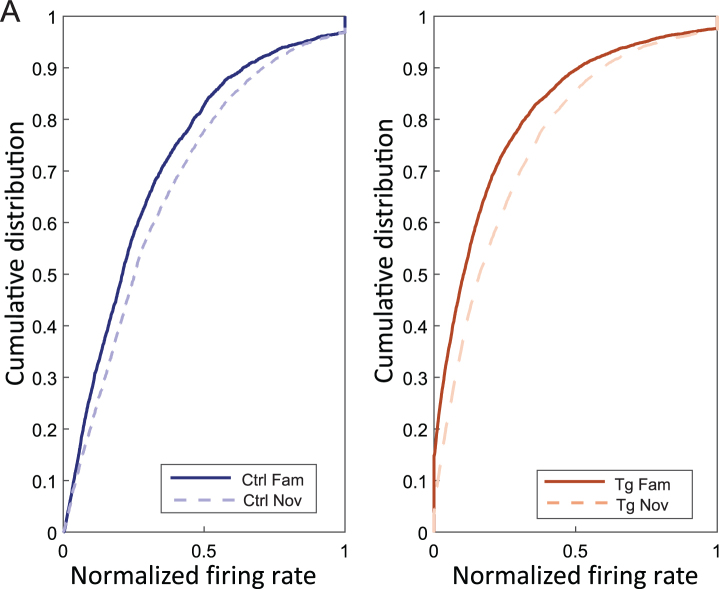
Increased normalized firing rate in novel environment. Panel A illustrates the cumulative distribution on the y-axis and the normalized firing rate on the x-axis in familiar and novel environments for both controls (left plot) and transgenics (right plot). The graph shows an increased firing rate in both groups in response to novel conditions.

In Fig. 4A, we showed the trend of cells with an increasing normalized firing rate from familiar to novel environments in both controls (fam: 0.27±0.02 Hz, nov: 0.30±0.02 Hz) and transgenics (fam: 0.18±0.01 Hz, nov: 0.22±0.01 Hz) [independent two-way ANOVA; group effect (F (1, 909) = 117.40, *p* < 0.001), session effect (F (1, 909) = 21.70, *p* < 0.001), group*session effect (F (1, 909) = 0.163, *p* = 0.687)]. Normalized firing rate values from 10000 bootstrapped samples were computed, all mean values from BS transgenic novel environments were higher than values from BS transgenic familiar environments (*μ* BS Tg nov > *μ* BS Tg fam = 0.04 Hz; *p* = 0) (Supplementary Figure 2B).

### AD animals showed an impaired precision of spatial coding

**Fig. 5:**
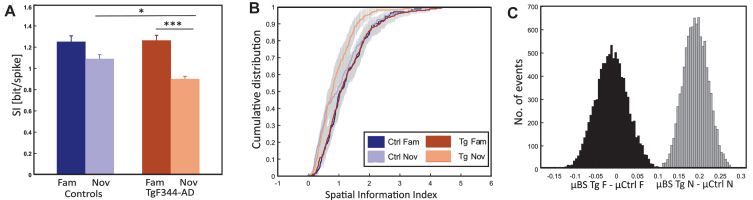
Spatial information differences between control and transgenic animals. Panel A depicts the comparison of the average spatial information index (y-axis) between groups (x-axis), revealing significantly impaired spatial coding in TgF344-AD rats compared to controls under novel conditions. Both groups exhibit a decrease in spatial information from the familiar to the novel environment; however, the drop is significant only in transgenics. Panel B illustrates the cumulative distribution (y-axis) of the spatial information index (x-axis) for all the original control and transgenic cell population (4 colored lines as in the description box) and along with bootstrapped transgenic cell population (2 grey shaded curves) in both conditions. Panel C depicts a histogram illustrating the bootstrapped distribution of the spatial information index. The y-axis represents the number of events, while the x-axis shows the sample differences between bootstrapped transgenics (BS Tg) and control animals in familiar (black-shaded) and novel environments (grey-shaded). The criteria for bootstrapping are described in the methods section. ^*^p < 0.05, ^***^p < 0.001.

The Spatial Information Index (SPI) determines the amount of information that a single spike conveys about the animal’s specific location.^31^ The activity of each cell on the linear track was calculated individually for each direction and sorted according to the higher values for statistical comparisons. In both controls and TgF344-AD rats, SPI values dropped from the familiar to the novel environment (Fig. 5A) (Ctrl cell no. = 174; fam: 1.25±0.05 bit/spike, nov: 1.08±0.03 bit/spike; Tg cell no. = 283; fam: 1.26±0.04 bit/spike; nov: 0.89±0.02 bit/spike). Spatial coding between controls and TgF344-AD in the novel condition was found to be significantly different by independent two-way ANOVA [group effect: F (1, 901) = 2.94, *p* = 0.08; session effect: F (1, 901) = 25.51, *p* < 0.001; group*session effect: F (1, 901) = 3.80, *p* = 0.05]. *Post hoc* analysis, using Tukey’s correction, revealed significant variation between groups in the novel condition (*p* = 0.05) and not in the familiar condition (*p* = 0.99). The within-group difference between conditions was significant in transgenics (*p* < 0.001) but not in controls (*p* = 0.20), as indicated by *post hoc* analysis.

Additional bootstrap testing was performed to account for a potential sample size effect in TgF344-AD (see methods). The overall representation of the cumulative distribution of the SPI for all the original control and transgenic cell populations along with the bootstrapped transgenic cell population in both conditions was shown in Fig. 5B. Average values from 10000 bootstrapped samples (BS) were calculated: *μ* BS Tg fam was 1.26 bit/spike, and the average difference between *μ* Ctrl fam and *μ* BS Tg fam was –0.01 bit/spike, whereas *μ* BS Tg nov was 0.89 bit/spike, and the average difference between *μ* BS Tg nov and *μ* Ctrl nov was 0.19 bit/spike. For the novel environment, all mean values from BS TgF344-AD were smaller than the mean values in controls (*μ* BS Tg fam < *μ* Ctrl fam = 3768, *p* = 0.31; *μ* BS Tg nov < *μ* Ctrl nov = 10000, *p* = 0; Fig. 5C).

**Fig. 6:**
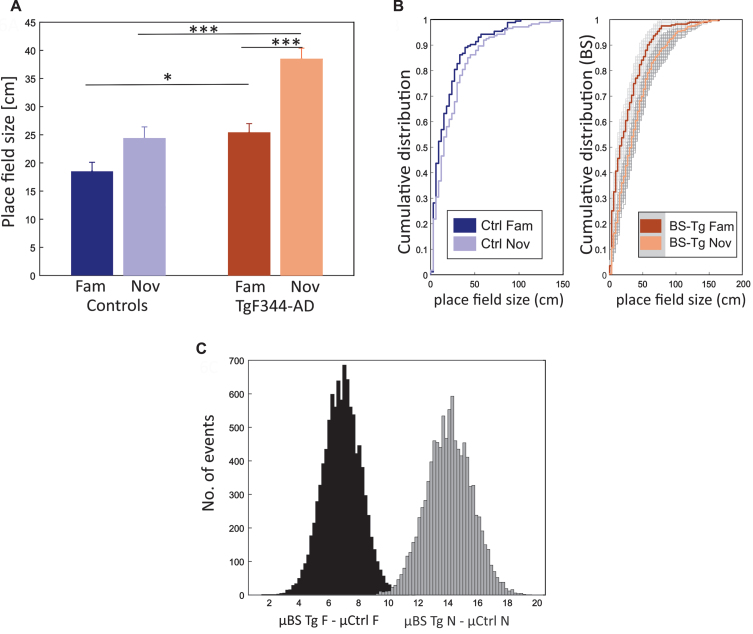
Place field size increase in novel environment within both groups. Panel A shows the comparison of Place Field size (PFs) (y-axis) between groups (x-axis), indicating a significant increase in PFs for TgF344-AD rats in both conditions compared to their control counterparts. Both groups show an increasing trend from familiar to novel conditions, with the change being significant only in the transgenics. Panel B consists of two plots, with the left plot displaying the cumulative distribution (y-axis) of PFs (x-axis) from controls in familiar and novel conditions. The right plot illustrates the cumulative distribution of PFs from bootstrapped transgenic samples within both conditions. Panel C shows a histogram illustrating the bootstrapped distribution of the PFs. The y-axis represents the number of events, while the x-axis shows the sample differences between bootstrapped transgenics (BS Tg) and control animals in familiar (black-shaded) and novel environments (grey-shaded). The criteria for bootstrapping are detailed in the methods section. ^*^p < 0.05, ^***^p < 0.001.

Another parameter representing space was Place Field size (PFs), which measured the percentage of the total area the place cell fired.^32^ Our data indicated that the subjects in the novel track exhibited an increased place field size. Figure 6A shows the length of PFs in familiar and novel environments. Place fields increased their size within the novel environment in both controls (cell no. = 174; fam: 18.5±1.61 cm, nov: 24.4±2 cm) and TgF344-AD (cell no. = 283; fam: 25.4±1.6 cm, nov: 38.5±1.9 cm). The increase was significantly higher in TgF344-AD rats compared to controls by Independent two-way ANOVA [group effect: F (1, 910) = 31.035, *p* < 0.001; session effect: F (1, 910) = 25.296, *p* < 0.001; group*session effect: F (1, 910) = 3.643, *p* = 0.05]. *Post hoc* testing using Tukey’s correction had shown a significance within group difference for PFs in TgF344-AD between familiar and novel conditions (*p* < 0.001), but not among controls (*p* = 0.195). The between-group comparison revealed a significant difference in place field sizes in both familiar (*p* = 0.05) and novel (*p* < 0.001) conditions.

Bootstrapped values were estimated (see methods): *μ* BS Tg fam was 24.4 cm, and the average difference between *μ* BS Tg fam and *μ* Ctrl fam was 6.9 cm, whereas *μ* BS Tg nov was 38.48 cm, and the average difference between *μ* BS Tg nov and *μ* Ctrl nov was 14.1 cm. For both familiar and novel environments, all BS transgenics were larger than the average value of controls (*μ* BS Tg fam > *μ* Ctrl fam = 10000, *p* = 0; *μ* BS Tg nov > *μ* Ctrl nov = 10000, *p* = 0; Fig. 6B, C).

### Direction-specific activity

Place cells develop directional specificity in an experience-dependent manner.^23,33^ Its formation is derived from the accessible cues within and outside the given environment.^22,23^ In our protocol, the level of environmental complexity was similar across the familiar and novel environment; however, we observed an increased proportion of unidirectional firing patterns of place cells in both conditions of controls compared to TgF344-AD rats. Also, the proportion of unidirectional cells in TgF344-AD within the novel environment was found to be significantly lower than in the familiar environment.

**Fig. 7:**
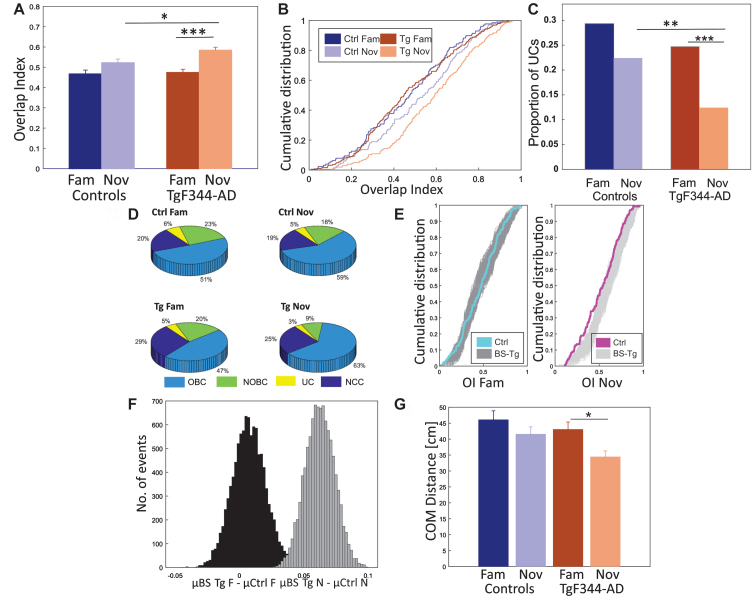
Higher proportion of unidirectional place cells in controls. In panel A, the increase in overlap index (OI) values (y-axis) from the familiar to the novel environment is depicted for both groups (x-axis), with a statistically significant difference observed only in TgF344-AD. The between-group comparison reveals a significantly greater number of overlapping direction-based representations in the novel environment within the transgenics. In panel B, the cumulative distribution (y-axis) of overlap index (x-axis) for both groups under familiar and novel conditions is presented. Panel C displays the proportion of unidirectional place cells (y-axis) over the total spatially selective cells between groups (x-axis). Transgenic animals express a significantly lower proportion of unidirectional cells in the novel environment than controls, while both groups exhibit similar ratios in familiar conditions. TgF344-AD rats show a significant drop in unidirectional cell proportions after exposure to the novel environment. Panel D presents a pie chart illustrating the categorization of all active cells based on their directional specificity within TgF344-AD and controls across both conditions. The criteria for cell classification are described in the methods section. Panel E shows the cumulative distribution (y-axis) of the overlap index (x-axis) among groups with bootstrap testing. The left and right panels display familiar and novel condition data, respectively. Blue and magenta lines represent control data, and shaded areas represent bootstrapped Alzheimer’s samples. In the familiar environment, control OI distribution matches bootstrapped samples, while in the novel track, control data displays lower values. Panel F presents a histogram displaying the bootstrapped distribution of the proportion of UCs. The y-axis represents the number of events, while the x-axis shows the sample differences between bootstrapped transgenics (BS Tg) and control animals in familiar (black-shaded) and novel environments (grey-shaded). The criteria for bootstrapping are described in the methods section. In panel G, the average center of mass (COM) distances (y-axis) between place fields in both directions are depicted across the groups (x-axis). Controls consistently display greater COM distances in both conditions. Within the transgenic group, there is a higher COM distance in the familiar condition and significantly lower values in the novel environment. ^*^p < 0.05, ^**^p < 0.01, ^***^p < 0.001, ^****^p < 0.0001.

For the overlap index quantification (OI, see methods), we selected only place cells with firing patterns in both directions. Independent two-way ANOVA comparison of resulting OI scores returned a significant difference between the groups [group effect: F (1, 646) = 4.134, *p* = 0.04; session effect: F (1, 646) = 23.564, *p* < 0.001; group*session effect: F (1, 646) = 2.609, *p* = 0.10]. The OI distribution tended to increase from the familiar to the novel environment within both groups but was significant only in TgF344-AD [*Post hoc* test using Tukey’s correction (Ctrl; *p* = 0.15) (Tg; *p* < 0.001)] (Fig. 7A, B). There was also a significant difference in OI between TgF344-AD and control in novel conditions (*p* < 0.05) by *post hoc* test using Tukey’s correction.

We considered the place cells with firing peaks of at least 1.2 Hz in both directions and OI exceeding the value of 0.4 as overlapped bidirectional cells (OBC). Those cells that had a smaller OI than 0.4 were classified as non-overlapped bidirectional cells (NOBC). Cells fired only in one direction with a firing peak of at least 1.2 Hz were classified as unidirectional cells (UC), and all other cells were classified as non-classified cells (NCC). We merged NOBCs and UCs under the UC category since the NOBC firing peak was prominent only in one direction. Control animals exhibited a higher proportion of UCs in familiar (29%; approx. 84 cells) and novel (22%; approx. 70 cells) environments, and there was no significant difference between the conditions (Chi-squared test: *p* = 0.18) (Fig. 7C). The proportion of UCs in TgF344-AD was lower in familiar (25%, approx. 81 cells) and significantly (Chi-squared test: *p* = 0.0002) dropped in the novel environment (12%; approx. 117 cells) (Fig. 7C). While the groups did not differ significantly in the familiar environment (Chi-squared test: *p* = 0.33), between-group comparison showed significantly lower proportion of UCs in the novel environment in Tg rats (Chi-squared test: *p* = 0.006). Although we classified cells based on the selected overlap index value of 0.4, we also observed significance in the p-value distribution of UCs within the OI range of 0.2 to 0.8 (median p: 0.017, SD±0.025) in the between-group comparison in novel condition, and within the OI range of 0.2 to 0.7 (median p: 0.0022, SD±0.0915) in the within-group comparison of transgenics.

We further categorized the proportion of active cells in controls with respect to familiar (Fig. 7D; OBC: 51%, NOBC + UC: 29%, NCS: 20%) and novel (OBC: 59%, NOBC + UC: 23%, NCS: 19%) environments. There were no robust changes in the proportions of overall cells within the control group (Chi-squared test - *p*-value: OBC - 0.4, NOBC + UC - 0.2, NCC - 0.9). However, in TgF344-AD rats, we observed a more evident cell proportion rearrangement from familiar (Fig. 7D; OBC: 47%, NOBC + UC: 25%, NCS: 29%) to the novel (OBC: 63%, NOBC + UC: 12%, NCS: 25%) environment (Chi-squared test - *p*-value: OBC - 0.001, NOBC + UC < 0.001, NCC - 0.3).

Average OI values from 10000 BS were calculated (see methods): *μ* BS Tg fam was 0.54, and the average difference between *μ* BS Tg fam and *μ* Ctrl fam was 0.007, whereas *μ* BS Tg nov increased to 0.62, and the average difference between *μ* BS Tg nov and *μ* Ctrl nov was 0.049. In the familiar environment, only a portion of the BS from TgF344-AD exceeded the average value of controls. In contrast, in the novel environment, all BS from TgF344-AD followed the consistent pattern (*μ* BS Tg fam > *μ* Ctrl fam = 7055, *p* = 0.15; *μ* BS Tg nov > *μ* Ctrl nov = 10000, *p* = 0; Fig. 7E, F).

We also evaluated the directional spatial selectivity using place field size-independent parameters such as center of mass (COM) distances. We calculated the COM distance between place cells in two directions to estimate directional spatial selectivity in both groups. The average COM distances from all place cell peaks firing in both directions were evaluated (Fig. 7G). Controls showed a higher average COM distance in both familiar (46.10±3.02 cm) and novel (41.57±2.78 cm) conditions compared to TgF344-AD animals (fam: 43.06±2.29 cm, nov: 34.44±1.85 cm). Independent two-way ANOVA showed a significant difference in COM distances between groups [group effect: F (1, 649) = 4.275, *p* = 0.04; session effect: F (1, 649) = 7.154, *p* = 0.01; group*session effect: F (1, 649) = 0.691, *p* = 0.40] (Fig. 7G). *Post hoc* analysis, using Scheffe’s correction, showed a significant difference in COM distances between familiar and novel environments in TgF344-AD rats (*p* = 0.05). However, no significant difference was observed between control familiar and novel environments (*p* = 0.70).

**Fig. 8:**
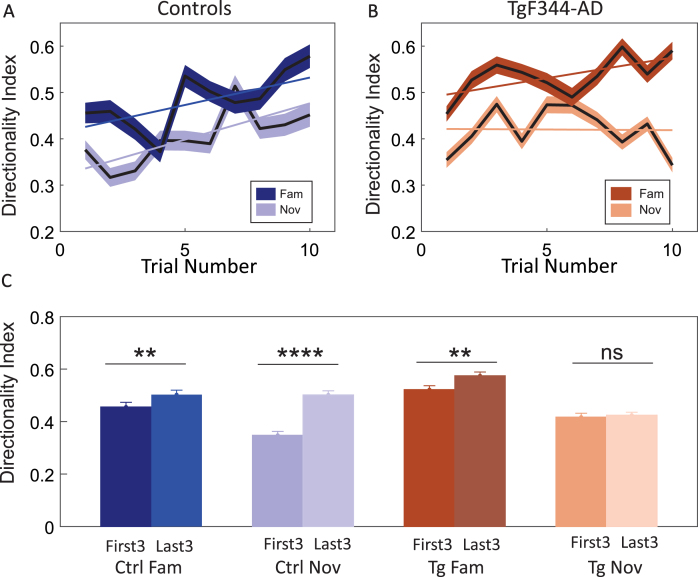
Evolution of directional firing rate specificity between groups. Panel A exhibits the directionality index (DI) (y-axis) across the time course of 10 trials (x-axis) in the control group. In both conditions, controls demonstrate a gradual increase in DI of firing rate over time. Panel B illustrates the directionality index (DI) (y-axis) across the time course of 10 trials (x-axis) in the TgF344-AD. TgF344-AD rats follow a similar gradual increase pattern in the familiar environment but not in the novel condition. Panel C features a bar graph comparing the DI averages (y-axis) from the first and last three trials in both groups (x-axis). Controls show a significant increase in DI from the first to the last sessions in both familiar and novel environments. In transgenics, a significant DI increase occurs only in the familiar environment and not in the novel environment. ^**^p < 0.01, ^****^p < 0.0001.

To quantify the evolution of place cell directionality across running trials in both environments, we evaluated the directionality index (DI) during each trial.^23^ DI measured the difference in firing rate within each running direction divided by the total firing rate in both directions. Controls exhibited an increase in DI in both the familiar and novel environments from the beginning to the end of the sessions (Fig. 8A). In contrast, TgF344-AD exhibited a rising pattern solely in the familiar environment, with no such trend observed in the novel condition (Fig. 8B). The data were assessed by multiple linear regression. The regression slope was statistically significant between Tg familiar and novel trials (β= –0.009, SE = 0.002, *p* = 0.001), while it was non-significant for controls between familiar and novel slopes (β= 0.003, SE = 0.004, *p* = 0.37).

Comparison between the DI of the first three trials (First3) and the last three trials (Last3) showed an increase within the familiar condition in both controls (first3- avg DI: 0.45±0.0013 Hz; last3- avg DI: 0.50±0.0014 Hz) and TgF344-AD (first3- avg DI: 0.52±0.0079 Hz; last3- avg DI: 0.57±0.0084 Hz) (Fig. 8C). This difference was significant in both control and TgF344-AD rats through *post hoc* analysis using P-holm correction (Ctrl: *p* = 0.04; Tg: *p* = 0.04). In contrast, the comparison between the first3 and last3 in the novel environment indicated a significant increase in DI in controls (first3- avg DI: 0.35±0.001 Hz; last3- avg DI: 0.50±0.0014 Hz) (*Post hoc*; P-holm correction: *p* < 0.001) but not in TgF344-AD (first3- avg DI: 0.41±0.007 Hz; last3- avg DI: 0.42±0.006 Hz) (*Post hoc*; P-holm correction: *p* = 1.000) (Fig. 8C). Independent two-way ANOVA showed a significant difference between the groups [group effect: F (1, 1801) = 9.189, *p* = 0.002; session effect: F (1, 1801) = 71.665, *p* < 0.001; trial effect: F (1, 1801) = 36.997, *p* < 0.001; group session trial effect: F (1, 1801) = 12.578, *p* < 0.001]. Overall, the statistics pointed towards the absence of place cell directionality development in TgF344-AD rats within novel conditions.

**Fig. 9:**
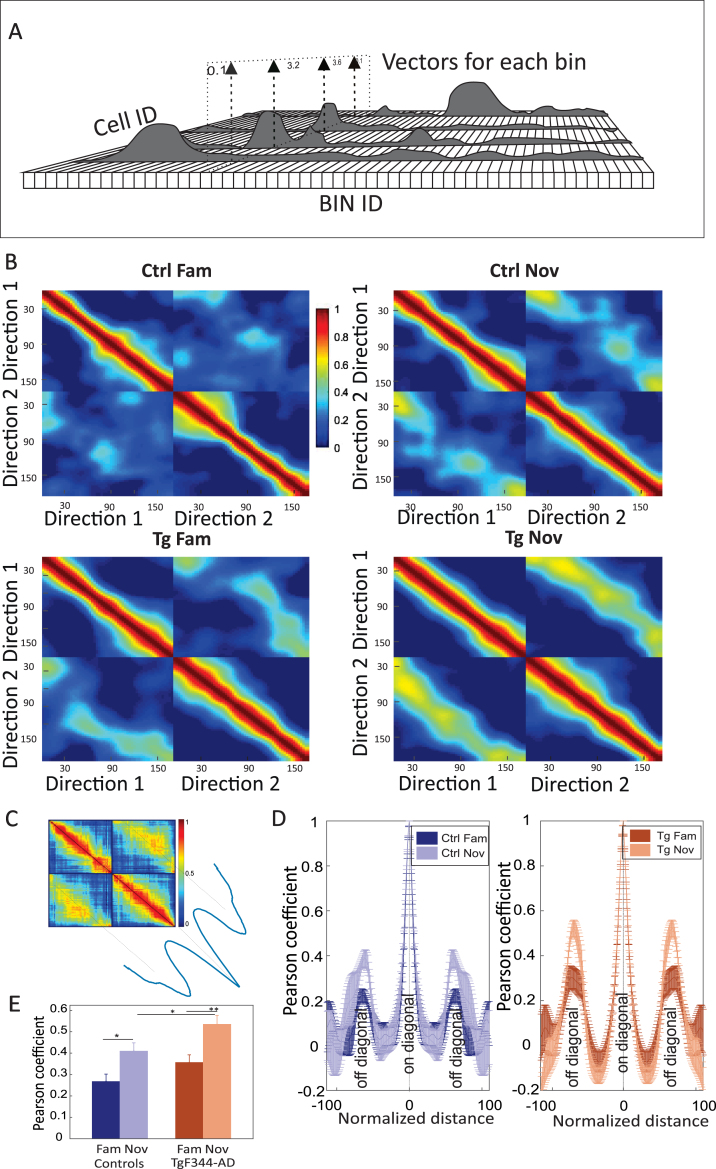
Population vector analysis across different directions in both groups. In panel A, a schema of population vector analysis is presented: Firing rates (z-axis) from spatial bins (x-axis) and cells (y-axis) form the population vector on the linear track. Panel B illustrates the population vector correlation matrix: each matrix represents the Pearson correlation between directions in the familiar environment (left side) and novel environment (right side). Panel C features a schema for the transposition of peak values from the diagonal cloud in panel B. Panel D involves the quantification of averaged peak values from the diagonal cloud of each correlation matrix (left-down or right-up quadrant in panel B). Both groups exhibit higher peaks in novel environments. Panel E provides a comparison of average peak values (y-axis) within each group and environment (x-axis). Significant differences are observed within both groups, but a significant between-group difference is found only in the novel environment condition. ^*^p < 0.05, ^**^p < 0.01.

A higher occurrence of bidirectional cells resulted in an increased similarity of the neural population concerning left and right-bound journeys. This dynamic nature can be quantified by population vector analysis across both directions of animal runs (Fig. 9A).^23,28,29^ The population vector correlation matrix depicted the similarity of a particular location in two different directions. The four panels in Fig. 9B, C represent the correlation matrices across neural population activity recorded during left and right direction runs, each forming 2×2 quadrants.

In both controls and TgF344-AD, we observed low correlation scores between left and right runs in familiar environments, indicating a low similarity between the respective codes. In the novel environment, the off-diagonal quadrants showed considerably higher population vector similarity in TgF344-AD than in the controls. Figure 9D depicts mean correlation values derived from the off-diagonal axis measured in the panels from Fig. 9B. Peak values from cross-directional population vector correlation comparisons between the groups showed significant differences, as assessed by independent two-way ANOVA [group effect: F (1, 16) = 10.398, *p* = 0.005; session effect: F (1, 16) = 23.116, *p* < 0.001; group*session effect: F (1, 16) = 0.297, *p* = 0.59]. Within-group *post hoc* comparisons showed a significant correlation increase of scores from familiar to the novel environment for both groups (*n* = 5 per group; Ctrl fam: 0.27±0.06 versus Ctrl nov: 0.41±0.07; *Post hoc* test (P-Holm correction): *p* = 0.03; Tg fam: 0.36±0.07 versus Tg nov: 0.54±0.08; *post hoc* test (P-Holm correction): *p* = 0.008) (Fig. 9E). Between-group population vector correlation comparison showed no significant differences between controls and TgF344-AD rats in the familiar condition (*post hoc* test by P-Holm correction; *p* = 0.15), but TgF344-AD showed increased correlation within the novel environment compared to the controls (*post hoc* test by P-Holm correction; *p* = 0.05) (Fig. 9E). This confirms the above-shown impairment of AD animals in refining the spatial code for the direction-specific context.

## DISCUSSION

The hippocampal representation of space enables the study of a wide range of mechanisms related to memory formation, retrieval, and other general aspects of neural information processing in the brain. Understanding its physiology simultaneously provides an unprecedented view into the pathophysiology of disease states affecting neural systems essential for spatial memory andnavigation.

In this report, we characterized the effects of rat Alzheimer’s-like phenotype on the spatial and directional tuning of hippocampal CA1 principal cell activity. We focused on the age level of 9–12 months, as at this age, the brain already exhibits clear beta-amyloid and hyperphosphorylated tau deposits. However, the extent of neurogenerative changes is still mild compared to older subjects used in earlier reports.^16,17,20,21^ Considering the signs of memory function deterioration and pathological hallmarks observed in the 9–12 months TgF344-AD animals, we regard this age group as a suitable model for studying the spatial impairment in AD.

Features of place cell spatial modulation are influenced by a variety of external and internal factors, namely the environment’s attributes such as size, shape, surrounding cues, or the animal’s direction of movement, running speed, etc. To better control their influence on hippocampal spatial coding, we used a linearized version of the environment that imposes more stereotypical behavior on subjects than widely used 2D arenas. Data from multiple traversals over identical locations at a relatively stable speed allows measuring a wider range of parameters, some of which reflect sustained plasticity of already formed neural representations.

Our analysis initially indicated a markedly increased place field size in transgenic subjects compared to controls, inversely mirrored in spatial information measures where the control animals yielded higher scores. This difference in place field size was detected in both familiar and novel environment conditions, but the contrast in spatial information content between groups was observed in the novel environment. The differences could not be attributed to variations in running speed; although animals moved, on average, faster on the familiar track, the speed scores were statistically indistinguishable across the groups. Within groups, the firing field sizes increased from familiar to novel conditions, but this novelty effect was significant only in the transgenic animals. This indicates that TgF344-AD subjects were impaired in the precision of coding the novel environment but still able to refine their spatial representation with experience, although not to the same extent as controls.

These findings, together with the spatial information index comparison, extend a previously reported study in 12–20 months old TgF344-AD.^20^ In contrast, spatial coding impairment in older TgF344-AD animals (18–20 months) in a familiar 2D open field was reported elsewhere, supposedly due to differences in experimental design, data analysis, and/or the chosen sex of the subjects.^22^

Place cell directional specificity is known to be linked to a variety of external and internal factors. Effects of goal locations, the amount of local and distal cues, or the extent of experience in task performance suggest complex mechanisms leading to the modulation of place cell firing in response to the animal’s movement direction.^22,33–35^ This phenomenon provides signs of contextual coding on top of the information about the animal’s position, allowing it to assess the ability to form complex representations of the external world. We analyzed the proportions of cells with and without directional tuning across both familiar and novel conditions. Although the directionality-related parameters were comparable between the groups in familiar conditions, the transgenic animals on the novel track were significantly less directionally specific. This finding was confirmed using four different methods. Two of these methods, the overlap index and population activity approach, were directly influenced by place field size, unlike the other two: the firing rate specificity and distances between the Centers of Mass (COM) of two distinct direction place fields. The impairment in the rate of map directional context-refinement was also confirmed during the novel environment session by comparing its initial and final map states. While this analysis revealed a remarkably steep increase in direction difference within the control group expressing a quick adjustment, a notably flat regression line was observed in the TgF344-AD rats. This indicates that the Alzheimer’s-like phenotype was impaired in the instantaneous formation of complex representations that contain both spatial and contextual components.^36,37^

To provide the corresponding evidence on a neuronal population level, we constructed activity vectors from individual cells firing across the entire available place cell populations, specific to each track and movement direction. Their correlation analysis thus provided a view of the degree of orthogonality between the population codes for each direction context within the given environment. The level of orthogonality in neural population coding is thought to have a direct connection to the capacity of episodic memory and the efficiency of spatial navigation.^38,39^ This is because increased orthogonality increases the number of unique activity patterns that can be encoded within a given neural network. The results confirmed the above findings, showing an impairment in the encoding of the directional context in the newly formed memory pattern in transgenic animals.

The discovered refinement failure seems relevant because it points to a mechanism that hasn’t been considered among factors leading to memory impairment in AD animal models so far. In more general words, it indicates that TgF344 AD subjects weren’t able to optimize their spatial code according to the stereotypical nature of the task. Instead, their representation reflected a stronger spatial component compared to controls, who were able to more effectively include the directional context, resulting in higher information content for their hippocampal maps. However, whether this mechanism directly impacts memory performance needs to be further clarified.

The diminished capacity to instantly encode the directional context into the spatial map indicates an impairment in optimizing the neural code for the stereotypical conditions imposed by the linear track environment. While the formation of spatial representations originates from a wide range of input stimuli, the observed effect in transgenic subjects could rise from impairments in sensory input information rather than disturbances at the hippocampal level.^40^ Additionally, since TgF344-AD animals showed similar directionality scores in familiar conditions and behaved indistinguishably during the initial vision check, we presume that their sensory processing was analogous to the controls. Therefore, the observed decline in the novel environment is more likely attributed to impairments in hippocampal information processing.

The exact nature of this deterioration is unclear. Changes in the synaptic plasticity and excitability of hippocampal principal cells that might delay or impair the formation of new memories, as well as their ongoing modifications, have been described in various AD animal models.^41,42^ Another mechanism may stem from disturbances in inhibitory circuits.^43^ Parvalbumin and somatostatin inhibitory synapses have been shown to stabilize place cell populations, facilitating the formation of distinct environmental representations within the hippocampus.^44^ Moreover, the general imbalance of inhibitory circuits and, consequently, their modulatory functions might directly affect the properties of neural oscillations that provide temporal routing mechanisms for network information processing.^45–47^ Dysfunctions in inhibitory circuits, along with related hippocampal oscillatory changes, have recently been described across different AD models.^48–50^

Another reason for the impaired complexity of the place cell code in TgF344-AD compared to controls could be the affected functional connectivity within hippocampal circuits, as well as on the hippocampal cortical level.^51,52^ On the input side, the entorhinal–hippocampal circuit, with its key projections from the superficial layers to the hippocampus, is among the earliest circuits involved in AD pathology.^53,54^ The question of which among the considered or other pathophysiological mechanisms underlies the presented effect remains to be elucidated.

## AUTHOR CONTRIBUTIONS

Athira Nataraj (Funding acquisition; Methodology; Resources; Validation; Writing – original draft; Writing – review & editing); Annu Kala (Methodology); Stephanie Lissette Proskauer Pena (Methodology); Karel Jezek (Writing – review & editing); Karel Blahna (Conceptualization; Formal analysis; Funding acquisition; Investigation; Methodology; Project administration; Supervision; Writing – original draft; Writing – review & editing).

## Supplementary Material

Supplementary Material

## Data Availability

The data supporting the findings of this study are available on request from the corresponding author.
