# Supplementary material for: Impaired Dynamics of Positional and Contextual Neural Coding in an Alzheimer’s Disease Rat Model
Source: J Alzheimers Dis. 2024 Aug 27;101(1):259–76. doi: 10.3233/JAD-231386 (PMC11612983; doi:10.3233/JAD-231386)
Supplement: Supplementary Material [file jad-101-jad231386-s001.pdf]

Supplementary Material

Impaired Dynamics of Positional and Contextual Neural Coding in an Alzheimer's Disease Rat Model

Supplementary Table 1. The following table details the number of runs for all animals in both control and TgF344-AD rats. We found no statistical significance between groups in both familiar [unpaired t-test; t (8) = -1.08; p = 0.31] and novel condition [unpaired t-test; t (8) = -1.48; p = 0.17].

Animals	Familiar	Novel
C1	19	08
C2	09	08
C3	09	14
C4	08	08
C5	08	08
Tg1	19	14
Tg2	12	12
Tg3	13	12
Tg4	13	10
Tg5	10	09

Supplementary Table 2. Both the TgF344-AD and control groups consisted of 5 animals, each with a varying number of recorded neurons. To eliminate the potential impact of sample size between the groups, a bootstrap analysis was performed (see methods). The table below provides the exact count of neurons for each animal.

Animals	No. of cells
C1	23
C2	56
C3	29
C4	43
C5	23
Tg1	61
Tg2	103
Tg3	29
Tg4	59
Tg5	31

Supplementary Figure 1

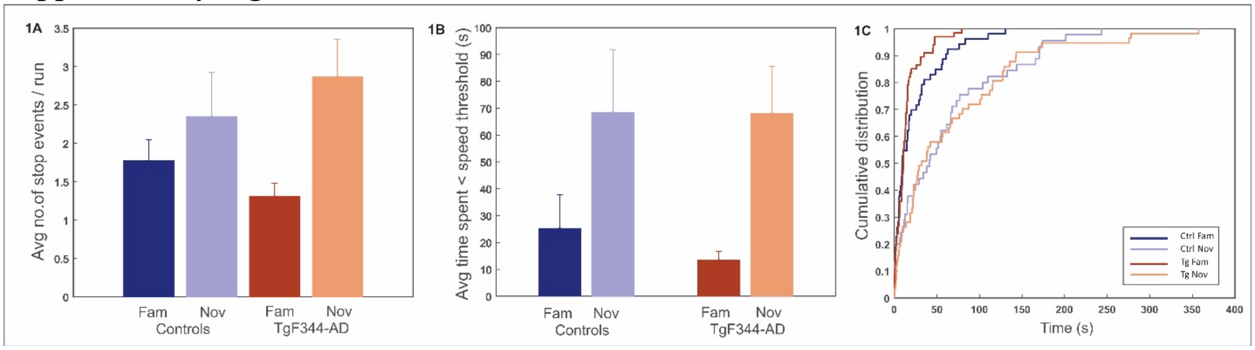

To eliminate the effect of pauses between runs on instantaneous speed and average firing rate calculations between controls and transgenics, we analyzed the average number of stops during a single run and the average time spent on the track during the pause (below the speed threshold) between groups. We observed no significant difference in the average number of stops per run between the groups, both in familiar (Ctrl: 1.77 ± 0.27; Tg: 1.31 ± 0.17) and novel (Ctrl: 2.35 ± 0.57; Tg: 2.87 ± 0.48) conditions [Independent two-way ANOVA, group effect: $F(1, 16) = 0.006$, $p = 0.94$] (A). Post hoc testing using Tukey's correction for group comparison also indicated no significant difference in both familiar ($p = 0.80$) and novel ($p = 0.68$) conditions. Comparison of the average time spent below the speed threshold between groups revealed non-significant variation on the familiar track (Ctrl: 25.29 ± 12.44; Tg: 13.45 ± 3.29) and on the novel track (Ctrl: 68.49 ± 23.39; Tg: 69.14 ± 17.44) [group effect: $F(1, 16) = 0.183$, $p = 0.67$] (B). Post-hoc testing using Tukey's correction for group comparison also showed no significant difference in both familiar ($p = 0.93$) and novel ($p = 1.00$) conditions. Panel C shows the cumulative distribution of the average time spent below the speed threshold for both groups in familiar and novel conditions.

Supplementary Figure 2

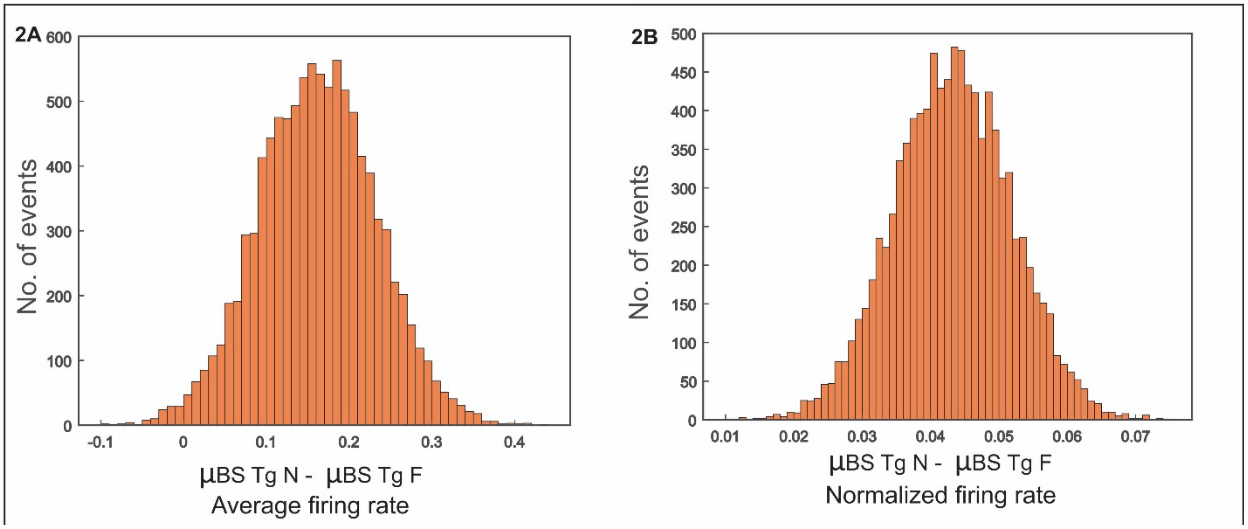

To eliminate the potential impact of sample size between the groups, we performed bootstrap testing for both average and normalized firing rates within the transgenic group, as it had the larger number of cells than controls. The histogram above illustrates the distribution of average firing rate (A) and normalized firing rate (B), with the y-axis representing the number of events and the

x-axis representing the difference between bootstrap (BS) transgenic samples from the novel to familiar environment. The criteria for bootstrapping are detailed in the methods section.
